# A Dinner to Dr. McKellops

**Published:** 1888-11

**Authors:** 


### EDITORIAL.

### A Dinner to Dr. McKellops.

"The San Francisco Dental Society gave a complimentary dinner at the Cosmos Club last Wednesday to Dr. H. J. McKellops, a distinguished dentist and scientist of St. Louis, who is making a short visit to this coast. Covers were laid for sixteen and many prominent members of the medical profession were present. Toasts were responded to by Dr. R. Beverly Cole, Dr. Simpson, and the guest of the evening. After spending several hours at the table the party broke up with the singing of "Auld Lang Syne." Dr. McKellops attended the midsummer high jinks of the Bohemian Club in the redwoods Saturday night, being the guest of Dr. Younger."

The above from a San Francisco paper will be read with pleasure by all who know Dr. McKellops, and to those who may not know him [they are few however] it is proper to say that the acts of recognition and appreciation, bestowed in this instance were well and rightly directed.

Few if any in the dental profession have done more to promote and establish the practical side of dentistry than Dr. McK. He travels from one end of the land to the other, to take part in association work; and he is never present that he does not present something of great practical interest and utility; generally it is something new, or if not this, it is a better and improved method of use, of something that has been before the profession, and in some instances for a long time; this in many instances is of far more value than the introduction of new things.

In him all true and right methods have a staunch supporter, but the shilly-shally and questionable methods have an implacable antagonist; it matters not who opposes, he stands for what he conceives to be right in practice.

---

BRONZING LIQUID.—Dissolve 10 parts of fuchsin and aniline purple in 100 parts of alcohol at 95° in a water-bath; add 5 parts of benzoic acid and boil for from five to ten minutes till of a bronze tint. Apply with a brush.—*Revue Professionnelle.*

